# MicroRNA-22 Regulates Hypoxia Signaling in Colon Cancer Cells

**DOI:** 10.1371/journal.pone.0020291

**Published:** 2011-05-23

**Authors:** Munekazu Yamakuchi, Shusuke Yagi, Takashi Ito, Charles J. Lowenstein

**Affiliations:** Department of Medicine, Aab Cardiovascular Research Institute, University of Rochester School of Medicine and Dentistry, Rochester, New York, United States of America; University of Bristol, United Kingdom

## Abstract

MicroRNAs (MiRNAs) are short, non-coding RNA that regulate a variety of cellular functions by suppressing target protein expression. We hypothesized that a set of microRNA regulate tumor responses to hypoxia by inhibiting components of the hypoxia signaling pathway. We found that miR-22 expression in human colon cancer is lower than in normal colon tissue. We also found that miR-22 controls hypoxia inducible factor 1α (HIF-1α) expression in the HCT116 colon cancer cell line. Over-expression of miR-22 inhibits HIF-1α expression, repressing vascular endothelial growth factor (VEGF) production during hypoxia. Conversely, knockdown of endogenous miR-22 enhances hypoxia induced expression of HIF-1α and VEGF. The conditioned media from cells over-expressing miR-22 contain less VEGF protein than control cells, and also induce less endothelial cell growth and invasion, suggesting miR-22 in adjacent cells influences endothelial cell function. Taken together, our data suggest that miR-22 might have an anti-angiogenic effect in colon cancer.

## Introduction

MicroRNAs (MiRNAs) are short non-coding RNAs (18–22 nt), which inhibit gene expression. Mature miRNAs are produced by the RNase III enzymes Drosha and Dicer, then incorporate into the RNA-induced silencing complex (RISC), and finally bind to the 3′-untranslated region (3′-UTR) of their target gene mRNAs, inhibiting their expression[1], [2]. It is believed that consecutive base pairing of at least 7 nucleotides between the miRNA sequence (seed sequence) and the miRNA recognition element (MRE) is necessary to repress protein translation[3], [4], [5], [6], [7]. In addition, some studies suggest that imperfect binding such as wobbles or bulges in the seed sequence inhibits protein translation [8], [9].

MiRNAs have a variety of physiological and pathological functions, including control of tumorigenesis[10], [11], [12]. The transcription factor most commonly mutated in cancer, p53, regulates a set of miRNAs. Activation of p53 increases miR-34a production, and over-expression of miR-34a induces cell cycle arrest, senescence and apoptosis. Another transcription factor linked to cancer, c-myc, regulates a separate set of miRNA. C-myc decreases the expression of several miRNAs including miR-22 in cancer cell lines[13]. Recent studies showed that miR-22 targets several proteins such as estrogen receptor a (ERa), c-Myc binding protein (MYCBP), Myc associated factor X (MAX), and PTEN, suggesting that miR-22 may be implicated in tumorigenesis. However the function of miR-22 in cancer cells remains unknown.

Hypoxia inducible factor 1 (HIF-1) is a heterodimeric transcription factor that regulates transcription of genes such as vascular endothelial growth factor (VEGF) and basic fibroblast growth factor (bFGF) [14], [15], [16]. HIF-1 is a heterodimer consisting of two subunits, HIF-1α and HIF-1β (ARNT). Hypoxia or hypoxia mimetics stabilize HIF-1α by inhibiting its prolyl hydroxylation. HIF-1 is involved in angiogenesis, invasion, metastasis, glucose uptake and metabolism in cancer cells[17]. Hypoxia in tumors can act as a trigger for angiogenesis to deliver increased oxygen to the cancer. HIF-1α expression is associated with poor prognosis in colorectal cancer and pancreatic cancer[17], [18], [19].

We now identify HIF-1α as a target for miR-22 in a colon cancer cell line. We find that miR-22 levels in human colon cancer are lower than in normal colon tissue. Since colon cancer specimens with lower miR-22 show higher VEGF expression, we hypothesize that miR-22 regulates hypoxia signaling in colon cancer cell lines.

## Results

### Expression of miR-22 in colon cancer

**Figure 1:**
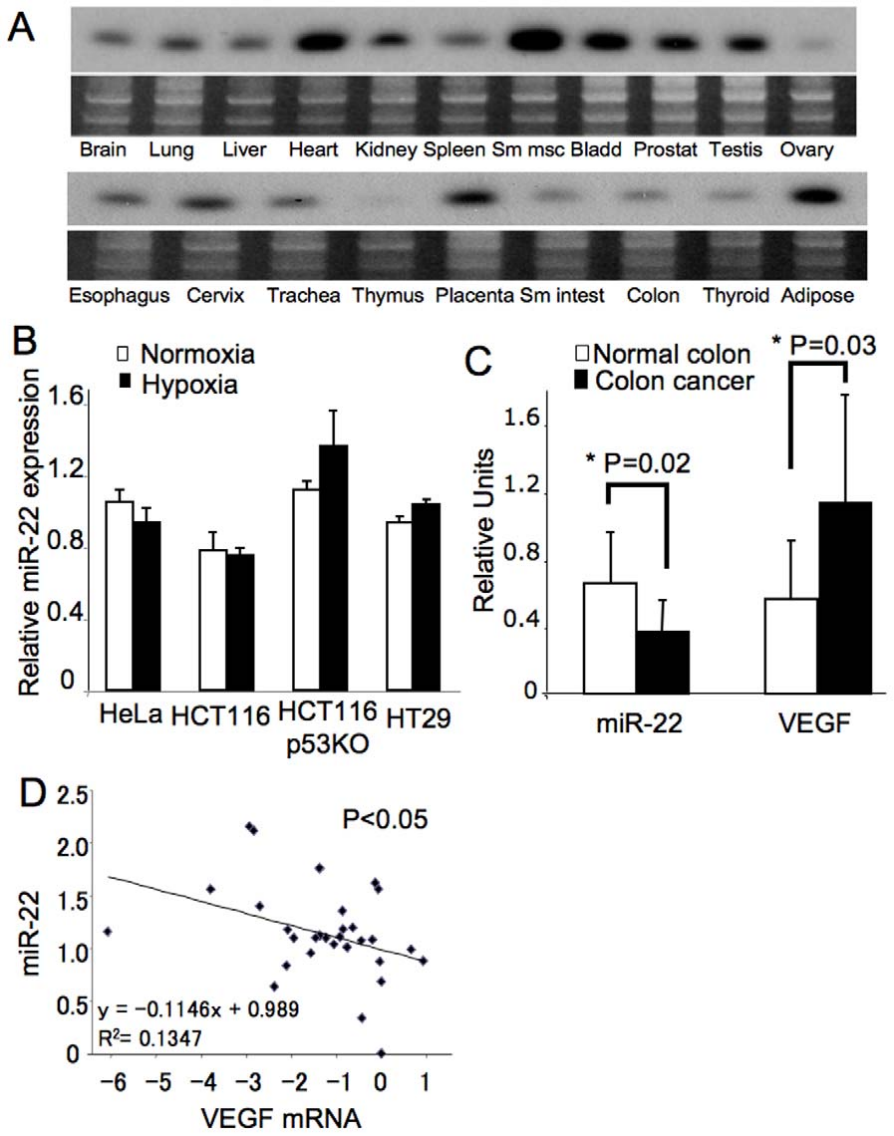
Expression of miR-22 in human cells, normal human colon tissue and human colon cancer. (A) MiR-22 expression in normal human tissues. A commercial membrane containing RNA from normal human tissues was probed for miR-22 using Northern analysis. (B) MiR-22 expression in cell lines. Cell lysates were probed for miR-22 by Northern blotting. (C) MiR-22 and VEGF expression in normal human colon tissue and human colon cancer. RNA was extracted from 9 normal human colon specimens (white) and from 9 human colon cancer specimens (black), and analyzed by qPCR for miR-22 and VEGF expression (n = 9± S.D.). Colon cancer specimens contain less miR-22 and more VEGF than normal colon specimens. (D) The association of miR-22 and VEGF in human colon cancer. Natural log transformation of relative ratio of RNA for miR-22 and VEGF was used for statistical analysis (n = 30).

We first used Northern blotting to measure miR-22 expression in human tissues, and found that miR-22 is expressed in most tissues, but relatively abundant in heart, smooth muscle, bladder, and adipose tissue (Fig. 1A). We next examined the expression of miR-22 in several cancer cell lines. We could detect miR-22 in three colon cancer cell lines, HCT116, HCT116 p53 KO and HT29, and also in an epithelial cancer cell line, HeLa (Fig. 1B). To examine the level of miR-22 in colon cancer, we measured miR-22 expression by qPCR in 9 human colon cancer specimens and 9 normal colon tissues from patients at The Johns Hopkins Hospital. Expression of miR-22 is lower in colon cancer specimens (P = 0.02) (Fig. 1C). Since we are interested in studying how microRNA regulate tumor angiogenesis, we also measured VEGF mRNA expression in the same samples and found that VEGF mRNA expression in colon cancer specimens is higher than that in normal colon specimens (P = 0.03) (Fig.1C). We also found that RNA levels of miR-22 and VEGF are negatively correlated (P<0.05) (Fig. 1D).

### HIF-1α is a target of miR-22

**Figure 2:**
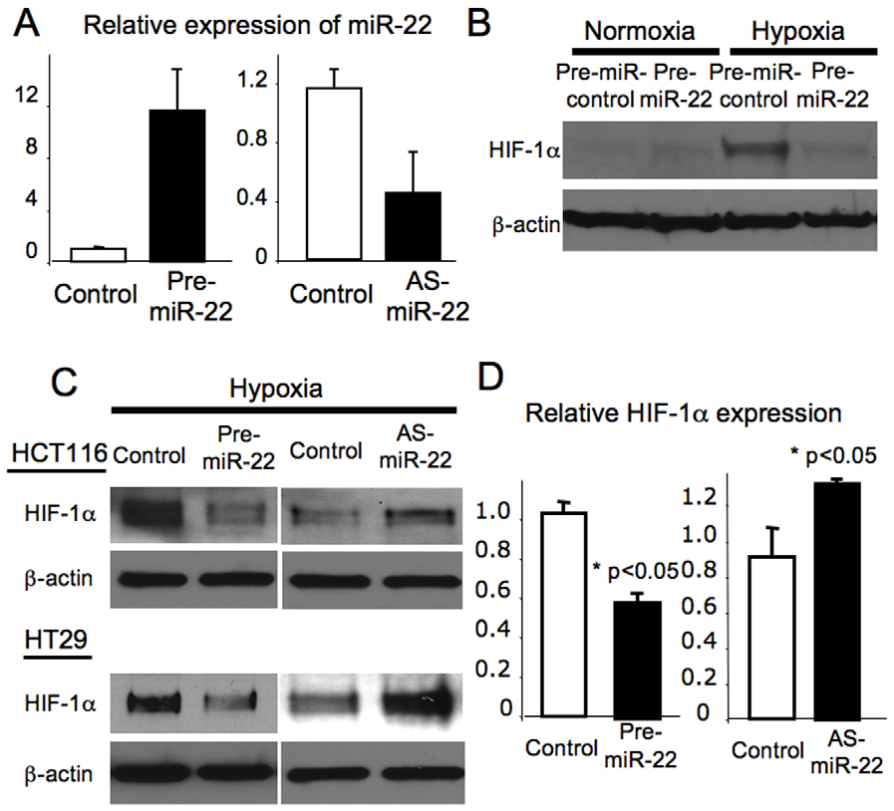
HIF-1α is a target of miR-22. (A) Alteration of miR-22 expression by transfection. HCT116 cells were transfected with pre-miR-22 or anti-sense-miR-22 or control oligonucleotides, and levels of miR-22 were measured by qPCR. (B) Over-expression of miR-22 inhibits HIF-1α expression. HCT116 cells were transfected with control oligonucleotides or pre-miR-22, and then exposed to normoxia or hypoxia for 16 h. Cell lysates were immunoblotted for HIF-1α. Hypoxia induces HIF-1α, but miR-22 suppresses HIF-1α. (C) Two colon cancer cell lines, HCT116 (two upper panels) and HT29 (two bottom panels), were transfected with pre-miR-22 or anti-sense-miR-22, exposed to hypoxia, and cell lysates were immunoblotted for HIF-1α. (D) Quantification by densitometry of immunoblotting for HIF-1α in HCT116 cells transfected as above (n = 3± S.D.).

Since HIF-1α is part of a major oxygen sensing pathway, we searched for potential miRNA that might control HIF-1α translation using computational analysis (Human miRNA Targets at the Memorial Sloan-Kettering Cancer Center Computational Biology web site http://cbio.mskcc.org/cgi-bin/mirnaviewer/mirnaviewer.pl), and found that the miR-22 seed sequence matches the 3′ UTR of HIF-1α (7 nucleotides matches including one wobble match). We explored how miR-22 regulates HIF-1 and hypoxia signaling using HCT116 colon cancer cells as an in vitro model of how tumor cells respond to hypoxia. To examine if miR-22 regulates HIF-1α protein expression, we transfected HCT116 cells with pre-miR-22 for over expression of miR-22 and with anti-sense-miR-22 for knockdown of miR-22. Transfection of pre-miR-22 into HCT116 increased miR-22 levels more than 10 fold (Fig. 2A). Knockdown of miR-22 by transfecting with anti-sense-miR-22 decreased miR-22 levels down to 40% (Fig. 2A). Hypoxia increased HIF-1α expression in HCT116, as expected (Fig. 2B). However, over-expression of miR-22 inhibited hypoxia-induced HIF-1α expression in HCT116 and HT29 (Fig. 2B–D). In contrast, knockdown of miR-22 enhanced HIF-1α expression under hypoxia (Fig. 2C–D). Over-expression of miR-22 did not alter the level of HIF-1β, the dimerization partner of HIF-1α (Figure S1). These studies show that endogenous miR-22 inhibits HIF-1α.

**Figure 3:**
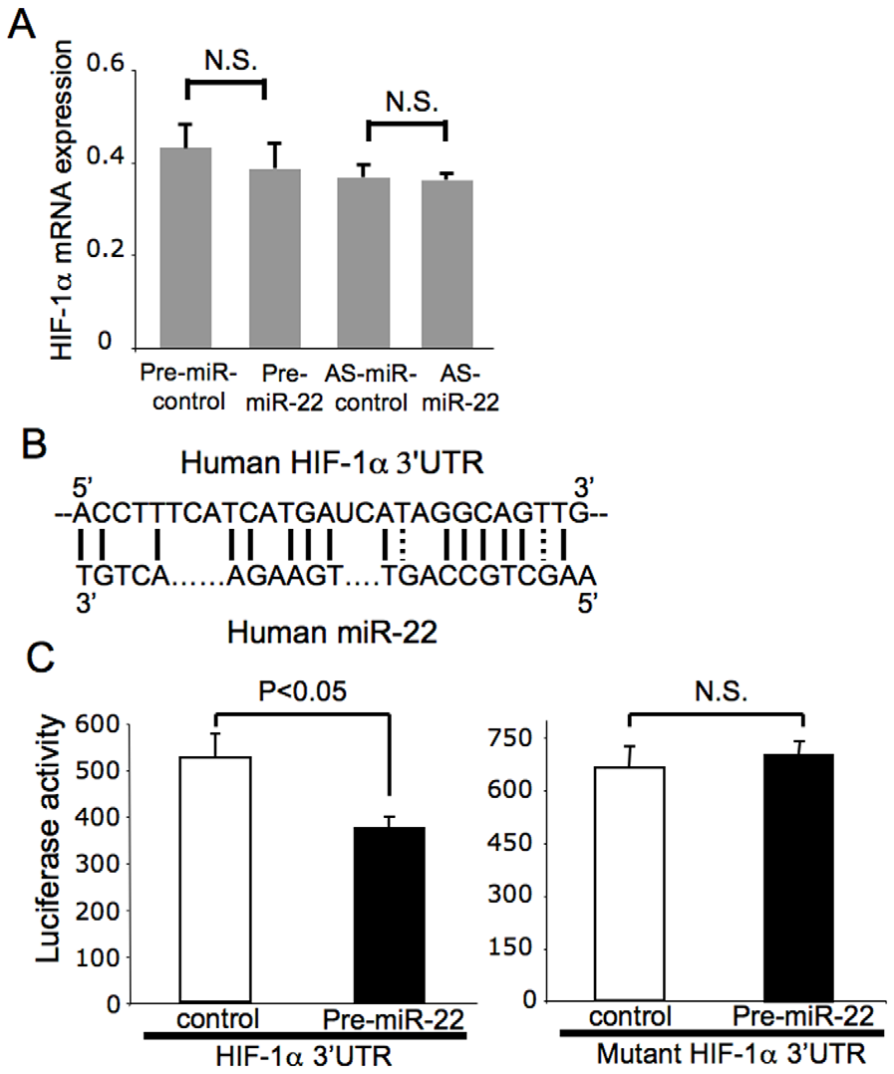
MiR-22 regulates HIF-1α by inhibiting its translation. (A) MiR-22 does not affect HIF-1α mRNA. HCT116 cells were transfected with Pre-miR-22 or anti-sense-miR-22 or control. Total RNA was harvested and analyzed for HIF-1α mRNA by qPCR (n = 3± S.D.). (B) Human HIF-1α 3′UTR contains a binding site for miR-22. (C) MiR-22 represses transactivation of HIF-1α 3′UTR. HCT116 cells were transfected with a luciferase reporter vector (MiRreport) containing the 3′ UTR of HIF-1α (left panel) or a mutated 3′UTR of HIF-1α (right panel) and with pre-miR-22 or pre-miR-control. Cells were harvested and luciferase activity was measured (n = 3± S.D.).

MicroRNA can regulate gene expression by suppressing translation. Over-expression or knockdown of miR-22 did not alter the expression of HIF-1α mRNA, which suggests that miR-22 regulates HIF-1α translation but not transcription (Fig. 3A). Human HIF-1α 3′ UTR has a potential binding site for miR-22 (Fig. 3B). To explore the mechanism by which miR-22 regulates HIF-1α, we made a luciferase reporter vector, which contains a fragment of the 3′ UTR of HIF-1α (extending after the stop codon from 0 to 401 bp) that includes a miR-22 binding site. We co-transfected HCT116 cells with this reporter vector and with pre-miR-22 or control. Over-expression of miR-22 decreased luciferase activity (Fig. 3C left). However, miR-22 does not affect the expression of luciferase with a mutated miR-22 binding elements (Fig. 3C right), suggesting that miR-22 inhibits HIF-1α expression via interaction with the 3′ UTR of HIF-1α.

### MiR-22 controls VEGF expression in HCT116

**Figure 4:**
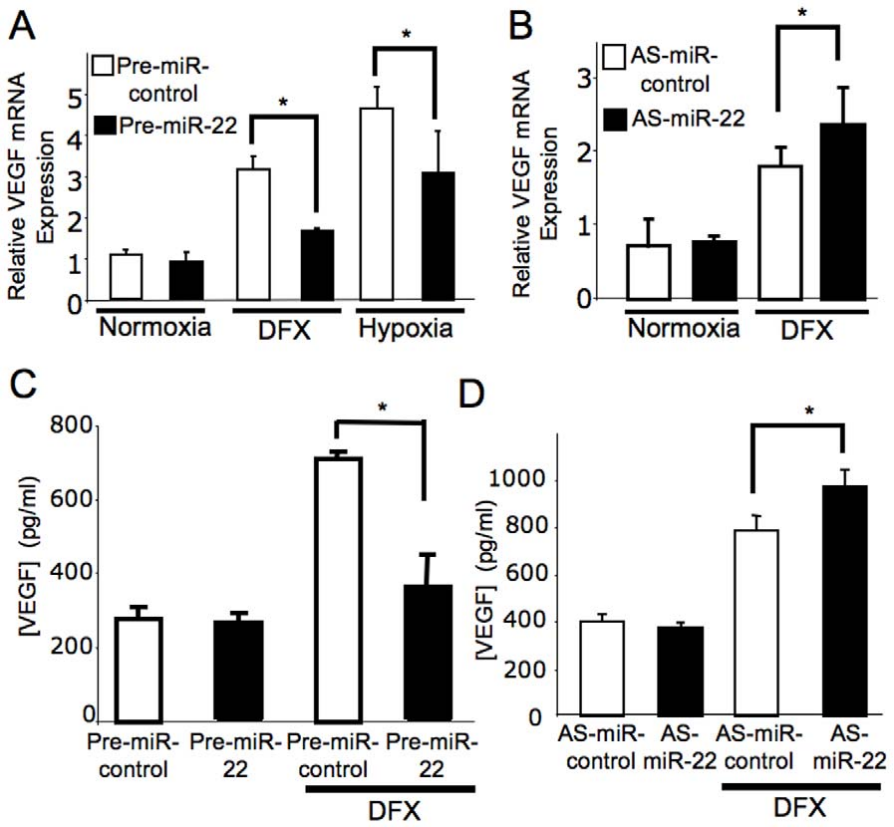
MiR-22 regulates VEGF expression and secretion. HCT116 were transfected with pre-miR-22 or anti-sense-miR-22 or control, and then exposed to normoxia or hypoxia for 16 h. The media was analyzed for VEGF by ELISA and RNA from cell lysates was analyzed for VEGF mRNA by qPCR (n = 3± S.D. *P<0.05) (A) Pre-miR-22 decreases VEGF mRNA. (B) Anti-sense-miR-22 increases VEGF mRNA. (C) Pre-miR-22 decreases VEGF protein. (D) Anti-sense-miR-22 increases VEGF protein.

To investigate the role of miR-22 in VEGF expression, we transfected HCT116 with pre-miR-22 or anti-sense-miR-22 or control, and then measured VEGF mRNA expression by qPCR. Hypoxia and the hypoxia mimetic desferioxamine (DFX) increased the expression of VEGF mRNA (Fig. 4A, white bars). Over-expression of miR-22 decreased hypoxia or DFX induced VEGF expression (Fig. 4A, black bars). Conversely, knockdown of miR-22 increased DFX induced VEGF expression (Fig. 4B). Next we measured the concentration of VEGF in the media. DFX increased the secretion of VEGF from HCT116 cells. Over-expression of miR-22 suppressed the secretion of VEGF. In contrast, knockdown of miR-22 enhanced VEGF release (Fig. 4C–D). Furthermore, we examined the effect of miR-22 upon expression of angiopoietin 2 (ANGPT2) and stem cell factor (SCF), because ANGPT2 and SCF are angiogenic growth factors that are regulated by HIF-1[20]. Hypoxia induced ANGPT2 and SCF, and over-expression of miR-22 inhibited hypoxia induced expression of ANGPT2 and SCF (Figure S2).

### MiR-22 in HCT116 regulates endothelial cell growth

**Figure 5:**
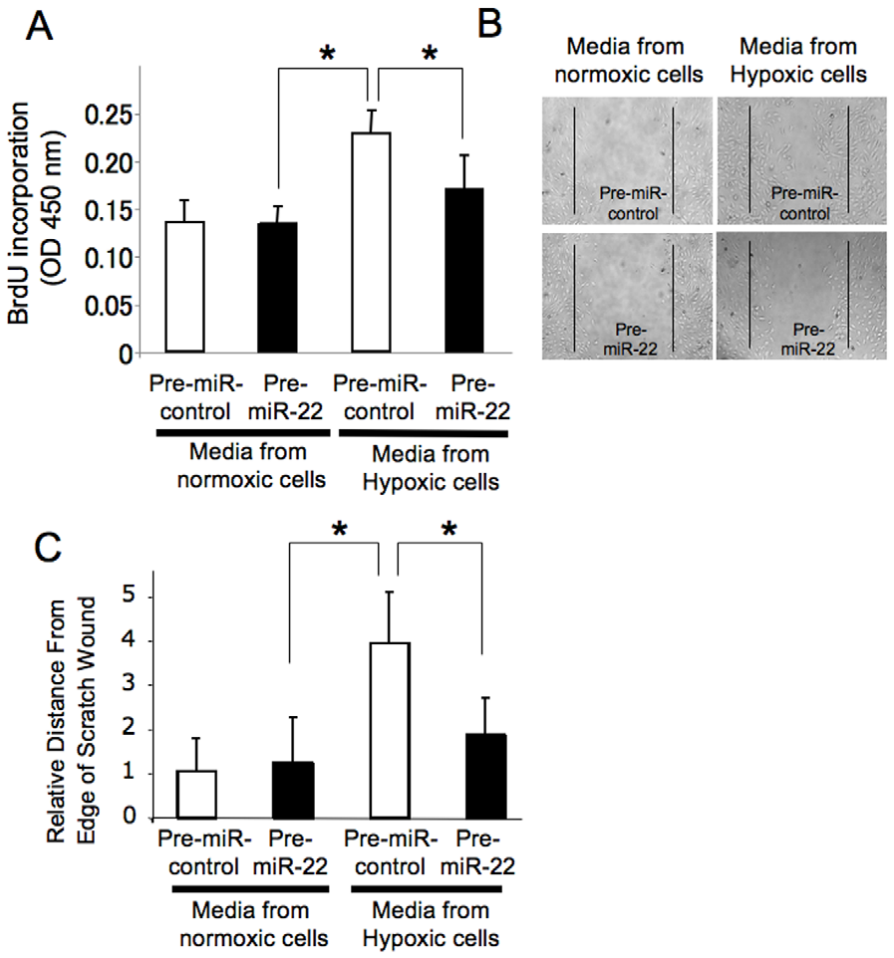
MiR-22 inhibits HCT116 production of VEGF and stimulation of endothelial cells. HCT116 cells were transfected with pre-miR-22 or control, and the media were collected. (A) HUVEC cells were grown in 12 well plates, the conditioned media were added, and the HUVEC were incubated for 3 days. Proliferation was monitored by BrdU incorporation assay (n = 5± S.D. *P<0.05). Pre-miR-22 decreases the ability of hypoxic treated cells to produce factors that stimulate endothelial proliferation. (B) HUVEC cells were scratched, the conditioned media were added, and photographed at 16 h. (C) Measurement of the distance covered by each wound relative to the control exposed with normoxia. (*P<0.05)

Since angiogenesis involves endothelial cell proliferation, we tested the effect of miR-22 upon HUVEC proliferation. We transfected HCT116 cells with pre-miR-22 or control, exposed the cells to normoxia or hypoxia, and harvested the media. We added this conditioned media to human primary endothelial cell (HUVEC), cultured for another 3 days, and then measured the proliferation of HVUEC by BrdU incorporation assay. There was no significant difference between media from normoxia cells transfected pre-miR-22 and media from normoxia cells transfected control (Fig. 5A). As expected, media from hypoxic cells transfected with control increased HUVEC proliferation. However, media from hypoxic cells transfected pre-miR-22 blocked this increase in proliferation (Fig. 5A).

Since angiogenesis also involves endothelial migration, we tested the effect of miR-22 upon HUVEC migration using the scratch wound healing assay. We added to HUVEC cells the conditioned media from HCT116 cells which had been transfected with pre-miR-22 or control and had then been exposed to normoxia or hypoxia. After 16 h we measured endothelial migration from the edge of the scratch wound. Media from HCT116 cells exposed to hypoxia increased endothelial migration (Fig. 5B–C). Over-expression of miR-22 in HCT116 slowed HUVEC migration (Fig. 5B–C). These data suggested that miR-22 in HCT116 affects endothelial biology, increasing proliferation and migration.

## Discussion

The major finding of this study is that miR-22 inhibits hypoxia signaling. MiR-22 is expressed in many cancer cells including the colon cancer cell line HCT116. Furthermore, miR-22 suppresses HIF-1α translation. Finally, miR-22 inhibits VEGF expression by suppressing HIF-1α expression. Since others have shown that c-myc limits miR-22 expression, tumors over-expressing c-myc might be expected to have lower levels of miR-22, higher levels of HIF-1α and VEGF. Therefore these data suggest that miR-22 may regulate tumor angiogenesis.

### Targets of miR-22

Individual miRNA can modulate expression of many genes. Several reports identify potential targets of miR-22 by using software algorithms, such as TargetScan, miRanda and PicTar, in combination with cellular studies. MiR-22 targets estrogen receptor a (ERa) and represses estrogen signaling in several breast cancer cell lines [21], [22]. MiR-22 also repressed c-Myc-binding protein MYCBP[23], c-Myc binding partner MAX[24] and tumor suppressor gene PTEN[25], [26], [27]. PPAR-alpha and BMP7 are also targets of miR-22[28]. We found that HIF-1α is a new target of miR-22. The 3′ UTR of HIF-1α includes a complementary sequence for miR-22 consisting of 7 nucleotides; this seed sequence contains one wobble binding nucleotide (G–A). Our biochemical data suggests that miR-22 directly regulates HIF-1α expression buy suppressing the translation of HIF-1α.

### Role of miR-22 in tumor angiogenesis

MiR-22 has unique roles in specific cell types. MiR-22 regulates the differentiation of a monocyte cell line[24]. MiR-22 regulates PPAR-alpha and BMP7 signaling pathways in human chondrocytes[28]. In cancer, the function of miR-22 is controversial. The mouse miR-22 gene is mapped to a cancer-associated genomic region, and the human miR-22 gene lies within a loss of heterozygosity region (LOH) in several cancer cells[23], [29], suggesting that miR-22 is involved in suppressing tumor growth. Ectopic expression of miR-22 inhibited the proliferation and colony formation of MCF-7 cells[23]. This tumor suppressor activity of miR-22 involves repression of MYCBP. However others have shown that that knockdown of miR-22 increases apoptosis rate in 16HBE-T cells[26]. The apparent contradiction between these two studies may be due to different cell lines or different methods for altering miR-22 levels. We found that miR-22 inhibits VEGF secretion, suggesting miR-22 may act as an anti-angiogenesis factor in colon cancer cell lines. Our human data support this idea: human specimens of colon cancer have lower levels of miR-22 and higher levels of VEGF, compared with normal human colon tissue. Our data reveal another mechanism through which miR-22 might act as a tumor suppressor: loss of miR-22 expression not only permits an increase in MYCBP but also an increase in HIF-1α.

### Hypoxia signaling and miRNA

Local tumor growth is limited by hypoxia: as the tumor expands, its center becomes hypoxic, inducing genes such as VEGF which trigger angiogenesis[30], [31], [32]. The HIF-1 heterodimer plays a critical role in hypoxic signaling in tumors[15], [33]. We and others have identified miRNA that control hypoxia signaling. We previously found that miR-107 inhibits HIF-1β[34]. Others have shown that miR-20b limits HIF-1α expression in lung adenocarcinoma and breast cancer cell lines[35], [36], [37]. The miR-17-92 cluster also modulates tumor growth by inhibiting HIF-1α expression. Our current study adds miR-22 to the list of miRNAs that regulate HIF-1 protein.

## Materials and Methods

### Cell Culture, Hypoxia exposure and Transfection

Human umbilical vein endothelial cells (HUVEC) were purchased from Lonza (Walkersville, MD). HUVEC (passage 2-5) were cultured in endothelial basal medium (EBM2) supplemented with growth factors (Lonza). HeLa and HEK293 (ATCC, Manassas, VA) were cultured in DMEM media (Invitrogen, Carlsbad, CA) supplemented with 10% fetal bovine serum (FBS). HCT116 (gift from Bert Vogelstein, The Johns Hopkins University School of Medicine) and HT29 (ATCC) were cultured in McCoy′s 5A media supplemented with 10% FBS. Desferrioxamine (DFX) and other reagents were obtained from Sigma (St Louis, MO). To expose cells to hypoxia, cells were cultured in Billups-Rotenburg chamber with 94% N2, 1% O2, and 5% CO2 on 37 C for 24 hours.

### Human Specimen

Human colon cancer specimens (n = 9) and paired non-cancerous normal colon specimen (n = 9) were obtained from patients at The Johns Hopkins Hospital, Baltimore, MD, with documented informed consent in each case. The collection and analysis of human colon cancer specimen were approved by The Johns Hopkins University School of Medicine Institutional Review Board. Patients undergoing surgery for colorectal cancers provided written consent to donate tissue for analysis.

### Transfection

Precursor miRNAs and anti-sense oligonucleotides for miRNAs were from Applied Biosystems (Foster City, CA). For transfection of precursor miRNA, HCT116 cells were transfected with siPORT NeoFX (Applied Biosystems) with precursor miRNA 0–20 nM or with anti-sense miRNA 0–40 nM and harvested 48 hours later.

### Northern blotting

Total RNAs were extracted from cells using Trizol (Invitrogen). Human tissue RNAs were obtained from Applied Biosystems. Northern blotting for miR-22 was performed as described previously. Briefly, 10 µg of each RNA were loaded onto 15% TBU-gel (Invitrogen), transferred to nitrocellulose membrane, and hybridized with ^32^P-end-labeled probes specific for miR-22 at 42°C for 16 hours. The miR-22 probe, 5′-TAAAGCTTGCCACTGAAGAACT-3′ was synthesized by Integrated DNA Technologies; all other reagents were purchased from Applied Biosystems.

### Quantitative Real-Time PCR (qRT-PCR)

To analyze miRNA expression, TaqMan MicroRNA assays were used to quantify levels of mature miRNAs following the manufacturer's instructions. Briefly cDNA was synthesized from purified small RNA (10 ng) and performed Real-Time PCR by using iCycler iQ (BioRad). Expression levels were normalized to U6. The primers for miRNA RT-PCR and the PCR mix were purchased from Applied Biosystems. To detect mRNA expression, cDNA synthesis was performed using High Capacity cDNA synthesis kit (Applied Biosystems). The primers for human VEGF and ß-actin were purchased from Applied Biosystems.

### Luciferase Assays

A fragment of the 3′ UTR of HIF-1α (starting after the TGA stop codon and extending for 401 bp) containing the miR-22 response element was cloned into pMIR-REPORT luciferase vector (Applied Biosystems). Mutation of the miR-22 response element (5′-GTTGACGG-3′ → 5′-G*A*T*C*A*G*GG -3′) was made by QuikChange Site-Directed Mutagenesis kit (Stratagene). HCT116 cells were plated at 5×10^4^ cells per well in 24 well plates. Next day, pMIR-REPORT Luciferase vectors including 3′ UTR of HIF-1α and precursor miR-22 or scrambled oligonucleotides were transfected into cells using Lipofectamine 2000 (Invitrogen). Forty-eight hours after transfection, luciferase assays were performed using the dual luciferase reporter assay system (Promega).

### Western blotting

Cells were lysed in 0.4 ml of lysis buffer (50 mM Tris pH 8.0, 150 mM NaCl, 10 mM EDTA, 1% NP40, 20 mM NaF, 1 mM orthovanadate and protease inhibitor cocktail). Lysates were separated by electrophoresis, blotted to membrane and reacted with specific antibodies. Antibody to mouse HIF-1α was from Cell Signaling Technology (Danvers, MA). All other primary antibodies and appropriate secondary antibodies were from Santa Cruz.

### VEGF Concentrations

An ELISA was used to quantitate VEGF secreted from HCT116 cells into the media. HCT116 cells were incubated in 1 ml of media with 1% FBS in the absence (controls) or presence of DFX or under normoxia or hypoxia for 24 h at 37°C. The supernatants were assayed for VEGF production using the Human VEGF ELISA kit according to the manufacturer's protocol (R&D Systems).

### BrdU incorporation assay

HCT116 or HeLa cells were transfected with Pre-miR-22 or Pre-miR-control and cultured for 72 hours. BrdU was added to each wells and incubated for 4 hours. After fixing cells, the cells were incubated with anti-BrdU antibody for 1 hour, then with Peroxidase Conjugated Goat anti-Mouse IgG for 30 min. TMB Peroxidase Substrate was added to each wells. Read the plate using a spectrophotometer microplate reader set at a wavelength of 450 nm.

### Wound healing assay

HUVECs were seeded on 12 well plates and grown to confluent. A scratch was performed using a 1000 µl pipette tip and media change to condition media from HCT116 transfected with Pre-miR-control or Pre-miR-22. Images were captured at 16 h and the distances between the cells were measured.

### Statistical analysis

Data were expressed as the mean ± SD. Statistical comparisons were made between two groups with the t test and between multiple groups by ANOVA. A value of P<0.05 was considered significant.

## Supporting Information

Figure S1 **HIF-1β is not a target of miR-22.** Description: HCT116 cells were transfected with pre-miR-22 or pre-miR-control, and exposed to normoxia or hypoxia for 16 h. Cell lysates were immunoblotted for HIF-1a and HIF-1b. Over-expression of miR-22 inhibits HIF-1a expression, but not HIF-1b.
(TIF)

Figure S2 **MiR-22 regulates the expressions of angiogenic factors.** Description: HCT116 cells were transfected with pre-miR-22 or control, and then exposed to normoxia or hypoxia for 8 h. RNAs from cell lysates were analyzed for angiopoietin 2 (ANGPT-2), stem cell factor (SCF), and COX-2 mRNAs by qPCR (n = 3± S.D. *P<0.05) Over-expression of miR-22 decreased the expressions of angiogenic factors.
(TIF)

## References

[1] Bartel DP (2009). MicroRNAs: target recognition and regulatory functions.. Cell.

[2] Filipowicz W, Bhattacharyya SN, Sonenberg N (2008). Mechanisms of post-transcriptional regulation by microRNAs: are the answers in sight?. Nat Rev Genet.

[3] Lewis BP, Shih IH, Jones-Rhoades MW, Bartel DP, Burge CB (2003). Prediction of mammalian microRNA targets.. Cell.

[4] Hammell M, Long D, Zhang L, Lee A, Carmack CS (2008). mirWIP: microRNA target prediction based on microRNA-containing ribonucleoprotein-enriched transcripts.. Nat Methods.

[5] Friedman RC, Farh KK, Burge CB, Bartel DP (2009). Most mammalian mRNAs are conserved targets of microRNAs.. Genome Res.

[6] Krek A, Grun D, Poy MN, Wolf R, Rosenberg L (2005). Combinatorial microRNA target predictions.. Nat Genet.

[7] John B, Enright AJ, Aravin A, Tuschl T, Sander C (2004). Human MicroRNA targets.. PLoS Biol.

[8] Brennecke J, Stark A, Russell RB, Cohen SM (2005). Principles of microRNA-target recognition.. PLoS Biol.

[9] Baek D, Villen J, Shin C, Camargo FD, Gygi SP (2008). The impact of microRNAs on protein output.. Nature.

[10] Krol  J, Loedige I, Filipowicz W The widespread regulation of microRNA biogenesis, function and decay.. Nat Rev Genet.

[11] Kim VN, Han J, Siomi MC (2009). Biogenesis of small RNAs in animals.. Nat Rev Mol Cell Biol.

[12] Carthew RW, Sontheimer EJ (2009). Origins and Mechanisms of miRNAs and siRNAs.. Cell.

[13] Chang TC, Yu D, Lee YS, Wentzel EA, Arking DE (2008). Widespread microRNA repression by Myc contributes to tumorigenesis.. Nat Genet.

[14] Maxwell PH, Dachs GU, Gleadle JM, Nicholls LG, Harris AL (1997). Hypoxia-inducible factor-1 modulates gene expression in solid tumors and influences both angiogenesis and tumor growth.. Proc Natl Acad Sci U S A.

[15] Semenza GL (2003). Targeting HIF-1 for cancer therapy.. Nat Rev Cancer.

[16] Kaelin WG, Ratcliffe PJ (2008). Oxygen sensing by metazoans: the central role of the HIF hydroxylase pathway.. Mol Cell.

[17] Semenza GL (2009). HIF-1 inhibitors for cancer therapy: from gene expression to drug discovery.. Curr Pharm Des.

[18] Baba Y, Nosho K, Shima K, Irahara N, Chan AT HIF1A overexpression is associated with poor prognosis in a cohort of 731 colorectal cancers.. Am J Pathol.

[19] Rasheed S, Harris AL, Tekkis PP, Turley H, Silver A (2009). Hypoxia-inducible factor-1alpha and -2alpha are expressed in most rectal cancers but only hypoxia-inducible factor-1alpha is associated with prognosis.. Br J Cancer.

[20] Semenza GL Vascular responses to hypoxia and ischemia.. Arterioscler Thromb Vasc Biol.

[21] Pandey DP, Picard D (2009). miR-22 inhibits estrogen signaling by directly targeting the estrogen receptor alpha mRNA.. Mol Cell Biol.

[22] Xiong J, Yu D, Wei N, Fu H, Cai T An estrogen receptor alpha suppressor, microRNA-22, is downregulated in estrogen receptor alpha-positive human breast cancer cell lines and clinical samples.. Febs J.

[23] Xiong J, Du Q, Liang Z Tumor-suppressive microRNA-22 inhibits the transcription of E-box-containing c-Myc target genes by silencing c-Myc binding protein..

[24] Ting Y, Medina DJ, Strair RK Schaar DG Differentiation-associated miR-22 represses Max expression and inhibits cell cycle progression.. Biochem Biophys Res Commun.

[25] Bar N, Dikstein R R miR-22 forms a regulatory loop in PTEN/AKT pathway and modulates signaling kinetics.. PLoS One.

[26] Liu L, Jiang Y, Zhang H, Greenlee AR, Yu R miR-22 functions as a micro-oncogene in transformed human bronchial epithelial cells induced by anti-benzo[a]pyrene-7,8-diol-9,10-epoxide.. Toxicol In Vitro.

[27] Poliseno L, Salmena L, Riccardi L, Fornari A, Song MS Identification of the miR-106b∼25 microRNA cluster as a proto-oncogenic PTEN-targeting intron that cooperates with its host gene MCM7 in transformation.. Sci Signal.

[28] Iliopoulos D, Malizos KN, Oikonomou P, Tsezou A (2008). Integrative microRNA and proteomic approaches identify novel osteoarthritis genes and their collaborative metabolic and inflammatory networks.. PLoS One.

[29] Calin GA, Sevignani C, Dumitru CD, Hyslop T, Noch E (2004). Human microRNA genes are frequently located at fragile sites and genomic regions involved in cancers.. Proc Natl Acad Sci U S A.

[30] Folkman J (1971). Tumor angiogenesis: therapeutic implications.. N Engl J Med.

[31] Ferrara N (2002). VEGF and the quest for tumour angiogenesis factors.. Nat Rev Cancer.

[32] Kerbel RS (2008). Tumor angiogenesis.. N Engl J Med.

[33] Semenza GL (2007). Hypoxia-inducible factor 1 (HIF-1) pathway.. Sci STKE.

[34] Yamakuchi M, Lotterman CD, Bao C, Hruban RH, Karim B P53-induced microRNA-107 inhibits HIF-1 and tumor angiogenesis.. Proc Natl Acad Sci U S A.

[35] Cascio S, D'Andrea A, Ferla R, Surmacz E, Gulotta E miR-20b modulates VEGF expression by targeting HIF-1 alpha and STAT3 in MCF-7 breast cancer cells.. J Cell Physiol.

[36] Lei Z, Li B, Yang Z, Fang H, Zhang GM (2009). Regulation of HIF-1alpha and VEGF by miR-20b tunes tumor cells to adapt to the alteration of oxygen concentration.. PLoS One.

[37] Hua Z, Lv Q, Ye W, Wong CK, Cai G (2006). MiRNA-directed regulation of VEGF and other angiogenic factors under hypoxia.. PLoS One.

