# Supplementary figures and images for: MicroRNA-22 Regulates Hypoxia Signaling in Colon Cancer Cells
Source: PLoS One. 2011 May 23;6(5):e20291. doi: 10.1371/journal.pone.0020291 (PMC3100326; doi:10.1371/journal.pone.0020291)

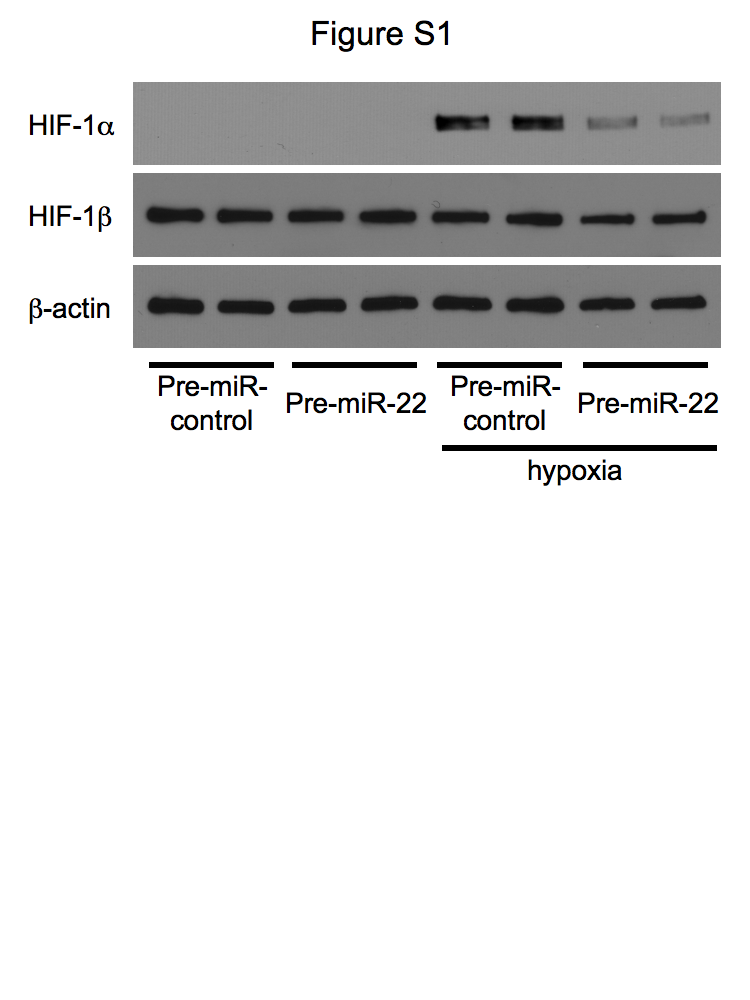

Supplement: Figure S1 — HIF-1β is not a target of miR-22. Description: HCT116 cells were transfected with pre-miR-22 or pre-miR-control, and exposed to normoxia or hypoxia for 16 h. Cell lysates were immunoblotted for HIF-1a and HIF-1b. Over-expression of miR-22 inhibits HIF-1a expression, but not HIF-1b. (TIF) [file pone.0020291.s001.tif]

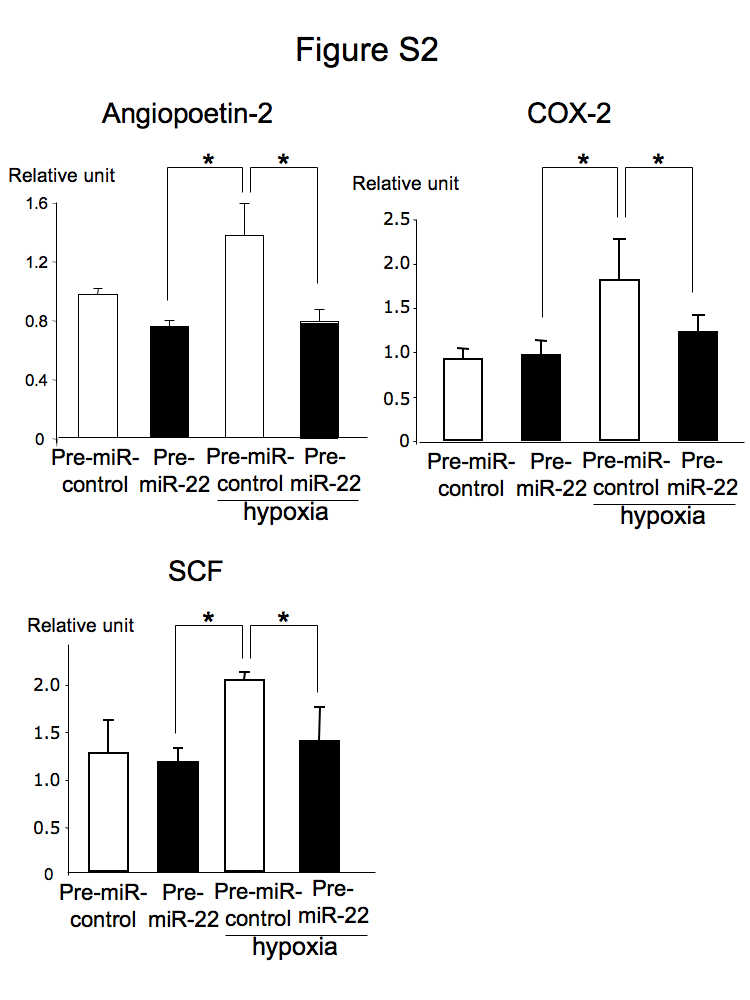

Supplement: Figure S2 — MiR-22 regulates the expressions of angiogenic factors. Description: HCT116 cells were transfected with pre-miR-22 or control, and then exposed to normoxia or hypoxia for 8 h. RNAs from cell lysates were analyzed for angiopoietin 2 (ANGPT-2), stem cell factor (SCF), and COX-2 mRNAs by qPCR (n = 3± S.D. *P<0.05) Over-expression of miR-22 decreased the expressions of angiogenic factors. (TIF) [file pone.0020291.s002.tif]
